# Dosimetric impact of deep inspiration breath hold (DIBH) technique in right-sided breast cancer radiotherapy: a retrospective analysis

**DOI:** 10.7717/peerj.20993

**Published:** 2026-03-23

**Authors:** Lu Jiang, Zhiwei Su, Zhao Jing, Chuan Sun, Zhibing Wu

**Affiliations:** 1 Department of Radiation Oncology, Zhejiang Hospital, Hangzhou, Zhejiang Province, China; 2 Zhejiang Key Laboratory of Geriatrics and Geriatrics Institute of Zhejiang Province, Affiliated Zhejiang Hospital, Hangzhou, Zhejiang Province, China; 3 Department of Oncology, Affiliated Zhejiang Hospital, Zhejiang University School of Medicine, Hangzhou, Zhejiang Province, China

**Keywords:** Right-sided breast cancer, Deep inspiration breath-hold, Body mass index, Body surface area, Free breathing, Lymph nodes, Liver, Heart, Right lung, Dosimetric analysis

## Abstract

**Background and Purpose:**

While deep-inspiration breath hold (DIBH) is widely used to reduce cardiac dose in left-sided breast cancer radiotherapy, its dosimetric benefits for right-sided cases remain underexplored. This study evaluated DIBH’s impact on target coverage and organs at risk (OARs) in right-sided breast cancer, and investigated associations between dose-volume changes, lung volume, and anthropometric indices.

**Methods:**

We retrospectively analyzed 33 patients with right-sided breast cancer treated using DIBH. Seventeen patients received whole-breast (WB) irradiation following post–breast-conserving surgery, and sixteen received chest wall (CW) plus internal mammary node (IMN) and supraclavicular (SC) irradiation post-mastectomy. Computed tomography (CT) scans acquired under free breathing (FB) and DIBH were used to generate separate treatment plans. Dose-volume parameters for target volumes and OARs were compared, and correlations between FB-to-DIBH changes and body mass index (BMI) and body surface area (BSA) were analyzed.

**Results:**

Compared with FB, DIBH significantly reduced doses to the right lung, heart, right coronary artery (RCA), and liver in both cohorts (all *P* < 0.05), while planning target volume (PTV) coverage remained unchanged. The maximum dose to the contralateral breast (Dmax) also decreased under DIBH, with reductions of 22.7% in the WB group (*P* = 0.02) and 37.76% in the CW+IMN+SC group (*P* < 0.001). Body mass index (BMI) showed a significant association with DIBH induced liver dose reduction in the WB cohort (liver mean dose, Dmean: *r* = 0.622, *P* = 0.008; liver volume receiving > =5Gy, V5Gy: *r* = 0.483, *P* = 0.05). In contrast, no meaningful correlations with dosimetric parameters were observed for BSA (all *P* > 0.05).

**Conclusion:**

DIBH effectively reduced OAR doses in right-sided breast cancer radiotherapy while maintaining target coverage. Patients with higher BMI derived greater hepatic dose sparing, suggesting that BMI-informed patient selection may enhance the clinical utility of DIBH.

## Introduction

Recent epidemiological data indicate that in 2020, breast cancer surpassed lung cancer to become the most prevalent cancer globally (Sung et al., 2021). Radiotherapy plays a pivotal role in breast cancer management, with conventional irradiation reducing the risk of local recurrence by approximately 50% and decreasing disease-specific mortality by nearly one-sixth (Darby et al., 2011). Regional nodal irradiation (RNI) can further enhance disease control by reducing locoregional recurrence rates by 30–40% (Whelan et al., 2015) and lowering the 5-year risk of distant metastasis by 5–8% (Poortmans et al., 2015). However, RNI inevitably increases radiation exposure to organs at risk (OARs), particularly the heart and ipsilateral lung, thereby elevating the long-term risk of radiation-induced cardiopulmonary toxicity.

The deep-inspiration breath hold (DIBH) is an effective respiratory management technique that has been widely implemented in left-sided breast cancer radiotherapy. Numerous studieshave demonstrated that DIBH significantly reduces radiation exposure to the heart and lungs, regardless of whether regional lymph nodes are included in the treatment volume (Stranzl & Zurl, 2008; Sakyanun et al., 2020; Falco et al., 2021; Ferdinand et al., 2021; Gaál et al., 2021). Based on this substantial clinical evidence, DIBH has become a standard-of-care technique for patients with left-sided breast cancer.

In contrast, the clinical utility of DIBH in right-sided breast cancer remains underexplored. The evidence suggests that DIBH may provide clinically meaningful reductions in ipsilateral lung and liver dose in selected right-sided patients (Conway et al., 2017; Borgonovo et al., 2022), and may offer additional benefit when regional lymph nodes are included in the target volume. Nevertheless, the dosimetric impact of DIBH under different treatment extents—particularly when the chest wall and regional nodal areas are irradiated—remains inadequately characterized, underscoring the need for further systematic evaluation.

Moreover, patients receiving concurrent chemotherapy or targeted therapies often exhibit reduced tolerance of radiation exposure to OARs. For example, anthracyclines (*e.g.*, doxorubicin) and taxanes substantially increase the risks of cardiotoxicity and hepatotoxicity (Friedrichs, Hölzel & Jänicke, 2002), while the combination of trastuzumab with anthracyclines and taxanes further amplifies cardiac risk *via* additive toxicity (de Azambuja et al., 2020). In such cases, the heart and liver become dual vulnerability organs, warranting enhanced dosimetric protection.

In this study, we conducted a comprehensive dosimetric analysis to evaluate the effects of DIBH on target coverage and OAR sparing in right-sided breast cancer patients. By stratifying patients according to the inclusion of regional nodal irradiation, we aimed to characterize the differential clinical benefits of DIBH. In addition, we investigated correlations between body mass index (BMI), body surface area (BSA), and critical OAR dose parameters, to help identify patient subgroups most likely to benefit from DIBH implementation.

## Materials and Methods

### General data

A total of 33 patients with right-sided breast cancer who received radiotherapy at Zhejiang Hospital between July 2021 and August 2024 were retrospectively enrolled and stratified into two groups (Table 1). Seventeen patients underwent breast-conserving surgery and received irradiation to the whole breast (WB group). Their ages ranged from 23 to 69 years, with a mean of 45.94 years and a median of 45 years. The remaining 16 patients received right chest wall (CW), internal mammary node (IMN), and supraclavicular (SC) nodal irradiation following radical mastectomy (CW+IMN+SC group), with ages ranging from 34 to 67 years (mean: 50.0 years; median: 49.5 years).

The study protocol was approved by the Zhejiang Hospital Ethics Review Committee, with approval number ZJHIRB-2025-048K. Informed consent was waived due to the retrospective nature of the study by the Zhejiang Hospital Ethics Review Committee. In this study, DIBH was performed using a voluntary breath-hold technique. Inclusion criteria were as follows: (1) use of a consistent breath-hold technique during simulation and treatment; (2) ability to sustain a single breath-hold for at least 20 s; (3) ability to repeat breath-holds at least six times during the simulation session. The exclusion criteria were as follows: (a) presence of a right breast implant; (b) cardiopulmonary insufficiency; (c) inability to cooperate with the treatment or to maintain a breath-hold for 20 s. Data for this retrospective analysis were collected and analyzed between May 1st and June 1st 2025.

### Computed tomography simulation and positioning

All patients were positioned supine with both arms raised and supported on a breast board, with a handgrip used to enhance stability. A vacuum immobilization cushion was applied to ensure consistent and reproducible positioning throughout imaging and treatment. In patients who underwent breast-conserving surgery, radiopaque lead markers were placed at three anatomical reference points: the *X*-axis marker at the center of the clavicle, the *Y*-axis at the nipple level, and the *Z*-axis along the anterior axillary line. For post-mastectomy patients, the *Y*-axis marker was placed three cm inferior to the sternal angle (carina), while the X- and *Z*-axis positions remained unchanged.

**Table 1:** Patient characteristics.

**Characteristics**	**WB Group (*n* = 17)**	**CW+IMN+SC Group (*n* = 16)**
Age (years)	45.9 ± 12.3(23–69)	50.0 ± 10.3(34–67)
Height (m)	1.59 ± 0.06	1.59 ± 4.59
Weight (kg)	56.3 ± 8.2	57.8 ± 8.2
BSA (m^2^)	1.56 ± 0.1	1.58 ± 0.1
BMI (kg/m^2^)	22.4 ± 3.5	22.9 ± 2.9
<18.5	2 (11.8%)	1 (6.2%)
18.5–24.9	10 (58.8%)	13 (81.3%)
≥25	5 (29.4%)	2 (12.5%)
Clinical stage		
I	11 (64.7%)	2 (12.5%)
II	5 (29.4%)	6 (37.5%)
III	1 (5.9%)	8 (50.0%)
Right diaphragmatic displacement (cm)	2.88 ± 1.34	3.25 ± 1.08

**Notes.**

Abbreviations WB whole breast CW+IMN+SC chest wall plus internal mammary node and supraclavicular regions BMI body mass index (BMI = weight/height^2^, kg/m^2^) BSA body surface area (BSA = 0.007184  × height^0.725^  × weight^0.425^)

Surgical bed scars from primary lesion were marked using lead wires to facilitate accurate target volume delineation. In post-mastectomy cases, a five mm tissue-equivalent bolus was applied to the chest wall surface to enhance skin dose coverage. Respiratory motion was monitored using an optical surface tracking system (Sentinel; C-RAD, Uppsala, Sweden) to ensured precise gating. Computed tomography (CT) simulation was performed using a Siemens scanner, acquiring axial images under both free breathing (FB) and DIBH conditions with a slice thickness of five mm. All image datasets were subsequently transferred to a third-party auto-contouring system for further processing and treatment planning.

### Target volume and organ-at-risk delineation

All target volumes were contoured by a single experienced radiation oncologist in accordance with the recommendations of ICRU Report 83. Clinical target volumes (CTV) and PTV were delineated separately on both FB and DIBH CT datasets. The left coronary artery (LCA) and right coronary artery (RCA) were manually contoured to ensure anatomical precision. OARs including the bilateral lungs, heart, and liver, were initially delineated using automated segmentation tools, followed by manual verification and adjustment when necessary to ensure contour accuracy.

### Treatment planning

All contoured image sets were transferred from the third-party auto-contouring platform to the Monaco treatment planning system (version 6.00.11; Elekta, Stockholm, Sweden). Using both FB and DIBH image datasets, treatment plans were generated by the same medical physicist utilizing fixed-field dynamic intensity-modulated radiotherapy (IMRT) with tangential beam arrangements and 6 MV flattening filter-free (FFF) photon energy. For each patient, the identical field arrangements were applied under both breathing conditions to ensure consistency in plan comparison. Dose calculations and optimization were performed using the Monte Carlo algorithm. For breast-conserving patients, PTV1 was defined as the tumor bed boost volume and prescribed 5,750 cGy in 25 fractions, while PTV represented the WB target volume and received 5,000 cGy in 25 fractions. For post-mastectomy patients, PTV encompassed the CW, IMN, and SC regions, with a prescription dose of 5,000 cGy in 25 fractions. Table 2 presents the dose constraints for OAR.

**Table 2:** Dose constraints for organs at risk.

**Structure**	**Dose Constraint**
Ipsilateral lung	*D*_mean_ < 15 Gy; *V*_20Gy_ < 30%; *V*_30Gy_ < 20%
Contralateral lung	*V*_5Gy_ < 20%
Contralateral breast	*D*_mean_ < 2 Gy; *D*_max_ < 5 Gy
Heart	*D*_mean_ < 5 Gy
Right coronary artery (RCA)	*D*_max_ < 20 Gy
Left coronary artery (LCA)	*D*_max_ < 20 Gy
Liver	*V*_5Gy_ < 20%

**Notes.**

Abbreviations: *D*_mean_ mean dose *V*_20Gy_ volume receiving ≥20 Gy *V*_30Gy_ volume receiving ≥30 Gy *V*_5Gy_ volume receiving ≥5 Gy *D*_max_ maximum dose

### Plan comparison

The following dosimetric parameters were evaluated: for the PTV, *D*_mean_, volume receiving ≥95% of the prescribed dose (*V*_95%_), dose to 2% of the volume (*D*_2%_), and dose to 98% of the volume (*D*_98%_); for the ipsilateral lung, *D*_mean_, *V*_5Gy_, volume receiving ≥20 Gy (*V*_20Gy_), volume receiving ≥30 Gy (*V*_30Gy_), and lung volume; for the contralateral lung, *V*_5Gy_ and *D*_mean_; for the contralateral breast, *D*_mean_ and *D*_max_; for the heart, *D*_mean_, *D*_max_, and *V*_5Gy_; for the LCA and RCA, *D*_mean_ and *D*_max_; and for the liver, *D*_mean_, *D*_max_, and *V*_5Gy_.

### Data analysis methods

Statistical analyses were performed using SPSS (version 19.0; IBM Corp., Armonk, NY, USA). Data are presented as mean ± standard deviation ($\bar {x}\pm s$). Normality was assessed using the Shapiro–Wilk test. Paired-sample t-tests and Pearson correlation analyses were applied to normally distributed variables, whereas the Wilcoxon signed-rank test and Spearman rank correlation were used for non-normally distributed data. *P* < 0.05 was considered statistically significant.

## Results

Table 3 presents the comparison of dose-volume parameters for PTV and OAR between the WB group (*n* = 17) and the CW+IMN+SC group (*n* = 16), Under both DIBH and FB conditions, no statistically significant differences were observed between the two groups in terms of PTV dosimetric parameters, including *D*_mean_, *V*_95%_, *D*_2%_, and *D*_98%_; all plans achieved the required target coverage. For the contralateral breast, DIBH resulted in a significant reduction in *D*_max_ in both cohorts. In the WB group, contralateral breast *D*_max_ decreased by 22.7% from FB to DIBH (*P* = 0.02), while in the CW+IMN+SC group, *D*_max_ decreased by 37.76% (*P* < 0.001). In contrast, the *D*_mean_ showed no significant difference between DIBH and FB in either group. The percentage reduction for each parameter was calculated as the difference between FB and DIBH values, divided by the FB value.

**Table 3:** Dosimetric comparison between WB and CW+IMN+SC groups under DIBH and FB. Reduction rate is calculated as (FB–DIBH)/FB.

Structure	Parameter	WB group (*n* = 17)				CW+IMN+SC group (*n* = 16)			
		DIBH	FB	Reduction	*P* value	DIBH	FB	Reduction	*P* value
PTV	*D*_mean_ (Gy)	53.0 ± 0.5	53.0 ± 0.6	0.0%	0.89	51.9 ± 0.5	51.9 ± 0.6	0.0%	0.796
	*V*_95%_ (%)	99.4 ± 0.3	99.4 ± 0.4	0.0%	0.94	98.6 ± 0.8	98.6 ± 0.7	0.0%	0.929
	*D*_2%_ (Gy)	59.5 ± 0.5	59.6 ± 0.8	0.2%	0.567	53.4 ± 0.5	53.4 ± 0.5	0.0%	0.918
	*D*_98%_ (Gy)	49.0 ± 0.3	49.0 ± 0.4	0.0%	0.70	48.2 ± 10.8	48.4 ± 0.8	0.4%	0.297
Right lung	Volume (cm^3^)	2,220.6 ± 283.1	1,347.2 ± 264.5	−64.8%	<0.001	2,108.2 ± 288.8	1,300.1 ± 158.4	−62.2%	<0.001
	*D*_mean_ (Gy)	10.0 ± 1.6	13.2 ± 1.5	17.3%	<0.001	13.3 ± 1.1	14.9 ± 0.8	11.1%	<0.001
	*V*_5Gy_ (%)	40.5 ± 5.1	48.1 ± 5.4	15.8%	<0.001	48.6 ± 4.5	54.5 ± 4.1	10.8%	<0.001
	*V*_20Gy_ (%)	20.1 ± 3.6	24.8 ± 3.1	19.0%	<0.001	24.9 ± 2.0	28.5 ± 1.6	12.6%	<0.001
	*V*_30Gy_ (%)	14.8 ± 3.1	19.0 ± 2.8	22.1%	<0.001	19.2 ± 2.3	22.3 ± 1.3	13.9%	<0.001
Left lung	*D*_mean_ (Gy)	0.9 ± 0.2	1.0 ± 0.3	8.3%	0.017	1.4 ± 0.3	1.4 ± 0.3	5.8%	0.040
	*V*_5Gy_ (%)	0.8 ± 1.2	1.5 ± 3.0	46.7%	0.916	2.1 ± 1.7	1.7 ± 1.7	−23.5%	0.438
Heart	*D*_mean_ (Gy)	1.7 ± 0.4	2.4 ± 0.8	29.7%	<0.001	1.9 ± 0.3	2.6 ± 1.3	25.7%	0.001
	*D*_max_ (Gy)	14.7 ± 9.0	20.8 ± 8.9	29.2%	0.011	13.8 ± 5.8	17.9 ± 8.3	22.8%	0.054
	*V*_5Gy_ (%)	2.1 ± 2.1	9.1 ± 7.9	76.9%	0.002	2.1 ± 1.4	6.9 ± 9.7	69.6%	0.002
LCA	*D*_max_ (Gy)	1.7 ± 0.6	2.2 ± 1.3	22.5%	0.039	2.0 ± 0.5	2.3 ± 0.6	14.3%	0.005
	*D*_mean_ (Gy)	1.4 ± 0.3	1.7 ± 0.7	20.3%	0.044	1.6 ± 0.3	1.8 ± 0.3	11.7%	0.003
RCA	*D*_max_ (Gy)	5.5 ± 3.2	10.0 ± 5.2	44.6%	0.006	6.3 ± 2.0	10.4 ± 5.5	39.4%	0.004
	*D*_mean_ (Gy)	3.1 ± 0.9	5.4 ± 2.7	43.3%	0.005	3.4 ± 0.9	4.9 ± 3.5	43.5%	0.002
Liver	*D*_mean_ (Gy)	1.0 ± 1.6	5.6 ± 4.4	81.4%	<0.001	2.6 ± 1.9	9.1 ± 4.1	72.0%	<0.001
	*D*_max_ (Gy)	25.5 ± 17.7	45.1 ± 15.3	43.5%	<0.001	39.8 ± 14.9	53.3 ± 1.4	25.4%	0.001
	*V*_5Gy_ (%)	3.1 ± 6.5	23.4 ± 18.1	86.8%	<0.001	10.2 ± 8.3	36.7 ± 13.5	72.2%	<0.001
Contralateral breast	*D*_max_ (Gy)	18.0 ± 12.3	23.3 ± 13.8	22.7%	0.02	15.0 ± 12.0	24.1 ± 17.2	37.76%	<0.001
	*D*_mean_ (Gy)	1.8 ± 0.7	1.9 ± 0.9	5.3%	0.43	2.3 ± 1.3	2.7 ± 1.5	14.8%	0.535

**Notes.**

Abbreviations: DIBH deep inspiration breath hold FB free breathing PTV planning target volume LCA left coronary artery RCA right coronary artery *V*_95%_ volume receiving ≥95% of the prescribed dose *D*_2%_ dose to 2% of the volume *D*_98%_ dose to 98% of the volume

### Dosimetric differences in lungs

In the WB irradiation group, the ipsilateral lung volume increased from 1,347.2 ± 264.5 cm^3^ under FB to 2,220.6 ± 283.1 cm^3^ under DIBH (*P* < 0.001), representing a 64.8% increase. In the CW+IMN+SC irradiation group, the volume increased from 1,300.1 ± 158.4 cm^3^ (FB) to 2,108.2 ± 288.8 cm^3^ (DIBH) (*P* < 0.001), representing a 62.2% increase. There was no statistically significant difference in the volume increase between the two groups.

**Figure 1:**
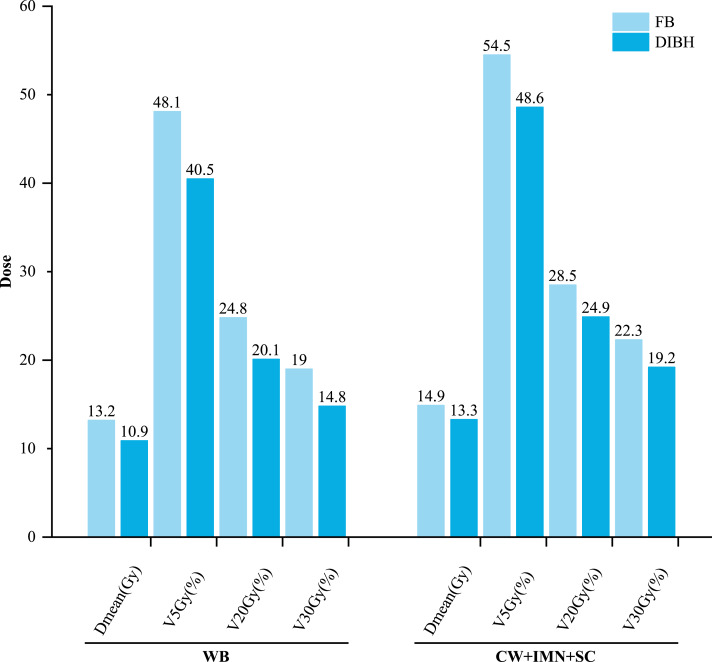
Comparison of right lung dose-volume parameters (D_mean_, V_5Gy_, V_20Gy_, V_30Gy_) between DIBH and FB in the WB and CW+IMN+SC groups. DIBH significantly reduced all parameters in the WB group and yielded greater dose reductions compared to CW+IMN+SC.

Figure 1 presents the dose-volume parameter comparison for the right lung under DIBH and FB. In the WB group, DIBH significantly reduced the right lung D_mean_, V_5Gy_, V_20Gy_, and V_30Gy_ compared to FB (*P* < 0.05). Furthermore, compared to the CW+IMN+SC group, the WB group demonstrated greater dose reductions under DIBH. In the CW+IMN+SC group, due to the larger irradiated volume, V_20Gy_ during FB was notably higher than in the WB group (28.5 ± 1.6% *vs.* 24.8 ± 3.1%). Two patients in this group had FB V_20Gy_ values exceeding 30%; DIBH successfully reduced V_20Gy_ to below 30%, with a maximum reduction of 20.1%.

For the contralateral lung, V_5Gy_ remained low (below 5.3% under FB), and DIBH showed limited impact on this parameter. No statistically significant differences were observed between FB and DIBH for left lung V_5Gy_ in either group (*P* > 0.05).

### Dosimetric differences in the heart

Both groups showed significant reductions in heart D_mean_ (WB group: *P* < 0.001; CW+IMN+SC group: *P* = 0.001), with the largest reduction observed in the WB group, decreasing from 3.7 Gy to 1.8 Gy—a 50.98% reduction. In the WB group, heart D_max_ and V_5Gy_ also significantly decreased by 29.2% (*P* = 0.011) and 76.9% (*P* = 0.002), respectively. In contrast, two patients in the CW+IMN+SC group exhibited increased D_max_ during DIBH, resulting in no significant group-level difference between DIBH and FB.

Both the LCA and RCA received reduced doses under DIBH. Notably, the RCA D_max_ dropped from 10.0 ± 5.2 Gy to 5.5 ± 3.2 Gy in the WB group (*P* = 0.006), and from 10.4 ± 5.5 Gy to 6.3 ± 2.0 Gy in the CW+IMN+SC group (*P* = 0.004).

### Dosimetric differences in the liver

**Figure 2:**
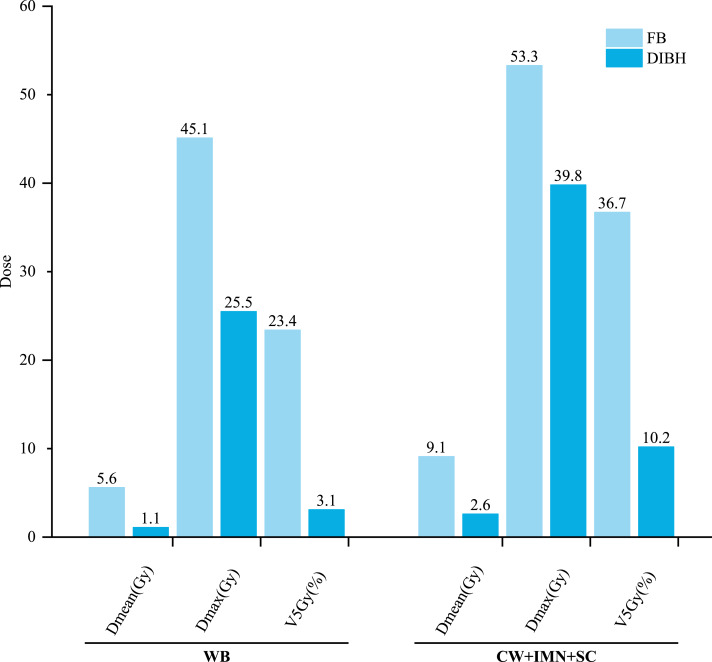
Comparison of liver dose-volume parameters (D_mean_, V_5Gy_, D_max_) between DIBH and FB in WB and CW+IMN+SC groups. Greater dosimetric benefit was observed in the WB group.

Figure 2 compares liver dose parameters between groups and shows that the WB group experienced greater reductions in liver exposure under DIBH compared to the CW+IMN+SC group. All 33 patients demonstrated varying degrees of reduction in liver D_mean_, V_5Gy_, and D_max_ under DIBH.

In the WB group, 12 out of 17 patients showed over 90% reduction in V_5Gy_, and five patients showed over 90% reduction in D_mean_. One patient’s liver D_max_ decreased from 47.6 Gy to 4.9 Gy, representing an 89.77% reduction. In the CW+IMN+SC group, four patients had V_5Gy_ and one patient had D_mean_ reductions exceeding 90%. The mean liver D_max_ declined from 53.3 ± 1.4 Gy to 39.8 ± 14.9 Gy (*P* < 0.001).

**Figure 3:**
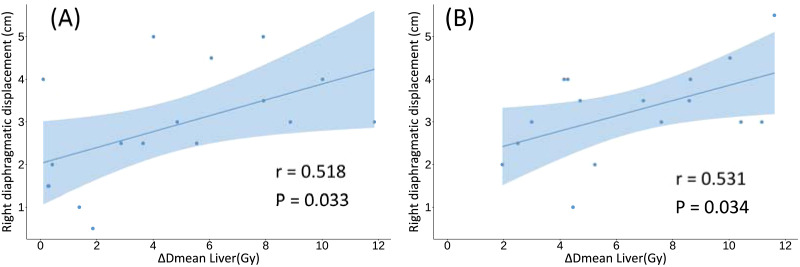
Correlation between changes in liver mean dose (Δ*D*_mean_ Liver) and right diaphragmatic displacement under DIBH. (A) WB group (*r* = 0.518, *P* = 0.033); (B) CW+IMN+SC group (*r* = 0.531, *P* = 0.034). Note: Δ*D*_mean_:Mean dose under free breathing minus mean dose under deep inspiration breath hold.

To further investigate whether the magnitude of liver sparing was associated with diaphragmatic movement during DIBH, Fig. 3 presents the correlation between Δ*D*_mean_ of the liver and right diaphragmatic displacement. The mean right diaphragmatic displacement during DIBH was 2.88 ± 1.34 cm in the WB group and 3.25 ± 1.08 cm in the CW+IMN+SC group. A significant positive correlation was observed in both cohorts (WB group: *r* = 0.518, *P* = 0.033; CW+IMN+SC group: *r* = 0.531, *P* = 0.034).

### Analysis of the correlation between BMI, BSA, and dosimetric parameters

Tables 4 and 5 summarizes the correlation analyses between BMI, BSA, and various dosimetric parameters. Correlation analyses were performed in the WB group (*n* = 17) and the CW+IMN+SC group (*n* = 16). BMI was calculated as weight divided by height squared (kg/m^2^), and BSA was calculated using the DuBois formula: BSA = 0.007184 × height^0.725^ × weight^0.425^.

**Table 4:** WB group: Correlation between BMI, BSA and dosimetric parameters, right-lung volume (*n* = 17).

Variable	Age	Volume R-Lung(FB)	ΔVolume R-Lung	Δ*D*_mean_ R-Lung	Δ*V*_20Gy_ R-Lung	Δ*D*_mean_ Heart	Δ*D*_max_ Heart	Δ*D*_mean_ Liver	Δ*V*_5Gy_ Liver	
BMI (*r*)	0.322	−0.458	−0.266	−0.143	−0.025	−0.227	−0.027	0.622	0.483	
BMI (*P*)	0.208	0.064	0.302	0.584	0.925	0.381	0.918	0.008	0.05	
BSA (*r*)	0.503	0.0	−0.189	0.236	0.198	−0.292	−0.267	0.258	0.198	
BSA (*P*)	0.039	0.999	0.468	0.361	0.446	0.255	0.3	0.318	0.446	

**Notes.**

Δ indicates the value under free breathing (FB) minus that under deep inspiration breath hold (DIBH).

**Table 5:** CW+IMN+SC group: Correlation between BMI, BSA and dosimetric parameters, right-lung volume (*n* = 16).

Variable	Age	Volume R-Lung(FB)	ΔVolume R-Lung	Δ*D*_mean_ R-Lung	Δ*V*_20Gy_ R-Lung	Δ*D*_mean_ Heart	Δ*D*_max_ Heart	Δ*D*_mean_ Liver	Δ*V*_5Gy_ Liver	
BMI (*r*)	0.358	−0.073	0.552	−0.358	−0.212	0.01	−0.053	−0.002	−0.03	
BMI (*P*)	0.173	0.789	0.027	0.174	0.43	0.97	0.844	0.994	0.912	
BSA (*r*)	0.234	0.014	0.326	−0.101	0.005	−0.003	0.075	0.055	0.048	
BSA (*P*)	0.384	0.959	0.218	0.709	0.986	0.991	0.783	0.839	0.859	

In the WB group, BMI showed no significant correlations with cardiac or lung dose parameters, except for a significant positive correlation with liver mean dose reduction (*r* = 0.622, *P* = 0.008) and a borderline correlation with liver *V*_5Gy_ reduction (*r* = 0.483, *P* = 0.05). BMI also demonstrated a non-significant trend toward a negative correlation with right-lung volume under FB (*r* =  − 0.458, *P* = 0.064). BSA exhibited a significant positive correlation with age (*r* = 0.503, *P* = 0.039) but was not significantly associated with any dosimetric endpoints.

In the CW+IMN+SC group, BMI was significantly correlated only with the change in right-lung volume (*r* = 0.552, *P* = 0.027), while no significant associations were observed with cardiac or liver dosimetric parameters. BSA showed no significant correlations with any dosimetric variables in this cohort.

## Discussion

Modern systemic therapies, particularly the prolonged use of endocrine agents such as aromatase inhibitors, have been associated with a significantly increased risk of cardiovascular disease in breast cancer survivors (Bovelli, Plataniotis & Roila, 2010; Amir et al., 2011). This concern underscores the importance of minimizing cardiac radiation exposure during radiotherapy. Notably, the risk of major coronary events increases linearly with mean heart dose (MHD), with each additional 1 Gy associated with a 7.4% increase in event incidence (Darby et al., 2013). The risk is particularly pronounced in patients requiring IMN irradiation (Hooning et al., 2007). In the present study, both patient cohorts demonstrated a statistically significant reduction in MHD of 0.7 Gy from FB to DIBH (*P* < 0.05), which is consistent with the findings of Essers et al. (2016), who reported a reduction of 0.4 Gy (*P* = 0.07).

Beyond reductions in MHD, the incidence of radiation-induced cardiac disease may also be associated with ischemic complications due to coronary artery irradiation (Gough et al., 2024) conducted a retrospective analysis of 14 patients receiving IMN irradiation after right-sided mastectomy and observed a 0.6 Gy reduction in the D_mean_ to the RCA (*P* = 0.002) and a 1.8 Gy reduction in the D_max_ (*P* = 0.002). In the present study, RCA dose reductions were more pronounced, with D_mean_ decreasing from 4.9 ± 3.5 Gy under FB to 3.4 ± 0.9 Gy under DIBH, and D_max_ decreasing from 10.4 ± 5.5 Gy to 6.3 ± 2.0 Gy (both *P* < 0.05). Similarly, Pandeli et al. (2019) reported a significant reduction in RCA D_max_ with DIBH (from 11.6 ± 7.2 Gy to 5.6 ± 2.9 Gy, *P* = 0.03), which is consistent with our findings (D_max_: 10.4 ± 5.5 Gy to 6.3 ± 2.0 Gy, *P* = 0.004). In the WB group, DIBH resulted in significant reductions in both D_mean_ and D_max_ for the RCA and LCA. Although LCA doses were relatively low under FB, the observed reductions remain clinically relevant under the principle of minimizing normal tissue exposure while maintaining adequate target coverage.

The magnitude of liver dose reduction observed in the present study was consistent with, and in some aspects exceeded, values reported in previous DIBH studies. Rice et al. (2015) reported reductions of approximately 50%–60% in low-dose liver exposure (*V*_5Gy_) with DIBH in breast radiotherapy, while Haji et al. (2019) demonstrated mean liver dose reductions of about 40%–60%, depending on treatment technique and field extent. More recently, Peters et al. (2022) and Lai et al. (2023) reported liver *V*_5Gy_ reductions ranging from 60% to 80% in right-sided breast cancer patients treated with DIBH, particularly when larger target volumes were included.In comparison, our results showed substantial liver sparing, with reductions in liver *D*_mean_, *D*_max_, and *V*_5Gy_ of 81.4%, 43.5%, and 86.8% in the WB group, and 72.0%, 25.4%, and 72.2% in the CW+IMN+SC group, respectively. These values fall within or above the upper range of previously reported reductions, supporting the robustness and credibility of our dosimetric findings. The more pronounced benefit observed in the WB cohort is consistent with prior studies indicating that liver sparing with DIBH is influenced by target extent and diaphragmatic excursion.

The risk of secondary lung cancer after radiotherapy in early-stage breast cancer patients has been shown to be closely related to lung radiation dose, with a particularly increased incidence among smokers (Grantzau et al., 2014). In the CW+IMN+SC group of the present study, the D_mean_ to the ipsilateral lung was significantly reduced by 11.1% (from 14.9 Gy under FB to 13.3 Gy under DIBH), comparable to the 13.4% reduction reported by Essers et al. (2016). Two patients exhibited right lung V_20Gy_ > 30% during FB, which was significantly reduced to 25.75% and 26.46% respectively after applying DIBH, successfully maintaining lung tissue exposure within clinically acceptable limits. Additionally, Mader et al. (2024) observed in their breast-only irradiation study that ipsilateral lung V_20Gy_ decreased from 14% to 11.5% (17.9% reduction), similar to our study’s 19% reduction. Previous studies have demonstrated that breast cancer patients receiving combined internal mammary and supraclavicular lymph node irradiation face significantly increased risk of radiation pneumonitis due to larger treatment volumes (Matzinger et al., 2010; Choi et al., 2016). In cases where ipsilateral lung dose-volume parameters exceed clinical constraints under FB, the application of DIBH may substantially improve ipsilateral lung dose distribution.

Correlation analysis between BMI, BSA, and dosimetric parameters revealed that BSA showed no significant association with any dose metric, probably because the DuBois formula—based solely on height and weight, does not capture differences in fat distribution or visceral organ displacement. In contrast, BMI demonstrated meaningful anatomical and dosimetric associations. In the WB cohort, higher BMI was associated with greater reductions in liver *D*_mean_ and *V*_5Gy_, supported by a positive correlation between Δ*D*_mean_ and right diaphragmatic displacement (*r* = 0.518, *P* = 0.033). These finding indicate that patients with higher BMI tended to achieve larger diaphragmatic excursions during DIBH, resulting in more pronounced inferior liver displacement and enhanced dose sparing. This pattern is consistent with left-sided DIBH studies demonstrating that patient-specific anatomical factors, including thoracic geometry, inspiratory capacity, and diaphragmatic excursion, significantly influence dose reduction (DellOro et al., 2019; Yamauchi et al., 2020; Wu et al., 2023). In the CW+IMN+SC cohort, BMI correlated only with changes in right-lung volume, whereas liver dose reduction was primarily associated with diaphragmatic motion (*r* = 0.531, *P* = 0.034). Although data specific to right-sided DIBH remain limited, previous studies have reported similar inter-patient anatomical variability influencing organ doses in right-sided radiotherapy (Lauffer et al., 2021). Collectively, these findings suggest that right-sided breast cancer patients with higher BMI and greater diaphragmatic excursion may derive the greatest hepatic benefit from DIBH.

While many existing studies incorporate lymph node irradiation, investigations focusing exclusively on breast-only irradiation remain relatively limited. The present study compared both treatment approaches and found that breast-only irradiation was associated with greater reductions in dose parameters for the ipsilateral lung, heart, coronary arteries, and liver compared with nodal irradiation. Although DIBH effectively reduced radiation exposure to organs at risk in right-sided breast cancer patients, its clinical implementation may be limited by prolonged treatment time, increased resource utilization, and greater patient burden. The development of predictive models based on FB anatomical characteristics and patient-specific parameters (*e.g.*, height, weight, and age) may facilitate more accurate identification of patients most likely to benefit from DIBH.

This study has several limitations. First, it was a single-center retrospective analysis with a relatively small sample size, which may introduce selection bias and limit the generalizability of the findings; validation in larger, multicenter cohorts is warranted. Second, only one irradiation technique—fixed-field IMRT—was evaluated; other delivery techniques may yield different OAR sparing patterns. Future multicenter prospective studies incorporating multiple irradiation techniques are needed to further validate these findings.

## Conclusion

In the context of right-sided breast cancer radiotherapy, the application of the DIBH technique in combination with optical surface-guided respiratory gating effectively reduces radiation exposure to the heart, lungs, liver, and contralateral breast. This reduction translates to a lowered risk of radiation-induced cardiac toxicity, radiation pneumonitis, and hepatic side effects, thereby demonstrating meaningful clinical value. Moreover, patients with higher BMI appear to derive greater hepatic dosimetric benefit from DIBH, suggesting that this technique should be preferentially considered for this subgroup when technically feasible.

## Supplemental Information

10.7717/peerj.20993/supp-1 Supplemental Information 1 Raw data

10.7717/peerj.20993/supp-2 Supplemental Information 2 STROBE checklist

## Additional Information and Declarations

### Data Availability

The following information was supplied regarding data availability: The raw dosimetric data is available in the Supplemental File.
